# A Systematic Review of Multifactorial Barriers Related to Breastfeeding

**DOI:** 10.3390/healthcare13111225

**Published:** 2025-05-23

**Authors:** Amparo Moret-Tatay, Marcelino Pérez-Bermejo, Adalberto Asins-Cubells, Carmen Moret-Tatay, María Teresa Murillo-Llorente

**Affiliations:** 1 Doctoral School, Catholic University of Valencia San Vicente Mártir, San Agustín 3, Esc. A, Entresuelo 1, 46002 Valencia, Spain; ammota@mail.ucv.es; 2 SONEV Research Group, Faculty of Medicine and Health Sciences, Catholic University of Valencia San Vicente Mártir, C/Quevedo No. 2, 46001 Valencia, Spain; mt.murillo@ucv.es; 3 Centro de Salud de L’Eliana, Departamento Arnau de Vilanova-Lliria, 46183 Valencia, Spain; asins_ada@gva.es; 4 Faculty of Psychology, Universidad Católica de Valencia San Vicente Mártir, 46100 Valencia, Spain; mariacarmen.moret@ucv.es

**Keywords:** breastfeeding, barriers, lactation, maternal health, infant nutrition, healthcare support

## Abstract

**Background/Objectives**: Breastfeeding is widely recognized as the best way to feed infants and has numerous health benefits for both mothers and infants. However, despite its well-documented benefits, breastfeeding rates remain lower than recommended in many parts of the world. This systematic review examines factors that create barriers for mothers trying to breastfeed, covering studies published between 2003 and 2025. **Methods**: A total of 18 studies were included in this systematic review, selected from the following databases: PsycINFO, MEDLINE, Academic Search Complete, Communication and Mass Media Complete, ERIC, SocINDEX, and CINAHL. Studies were selected based on predefined inclusion criteria, focusing on peer-reviewed articles that examined factors influencing breastfeeding practices. Data extraction and quality assessment were performed independently by two reviewers using standardized tools. The review analyzed personal, cultural, economic, and health-related barriers. **Results**: The analysis revealed multiple barriers to breastfeeding, categorized into personal, sociocultural, economic, and healthcare-related factors. Common challenges included a lack of counseling, latching difficulties, insufficient workplace support, and cultural misconceptions. The heterogeneity of study designs posed challenges in synthesizing the findings. **Conclusions**: More targeted policies and programs are needed to address these barriers and help mothers succeed in breastfeeding. Improving breastfeeding outcomes worldwide will require better healthcare, social support, and an understanding of cultural influences.

## 1. Introduction

Breastfeeding is endorsed by the World Health Organization [1] as the optimal feeding practice for infants. Exclusive breastfeeding for the first six months of life is not only recommended but also associated with numerous health benefits, including reduced infant mortality, improved immune function, and reduced risk of chronic disease. However, while the benefits are well documented, actual breastfeeding success rates do not always match recommendations. According to the WHO, less than 50% of infants worldwide are exclusively breastfed for the first six months, with significant regional variations. For example, increases in both exclusive and any breastfeeding at six months have been observed across regions and income groups, while increases in formula consumption have been observed in upper-middle-income countries [2]. These variations highlight the complex interplay of factors influencing breastfeeding practices.

In this scenario, many mothers may face significant challenges in their willingness to initiate or continue breastfeeding. Among these challenges, changing roles in women’s lives play a crucial role, particularly in relation to work [3]. In most societies, women are expected to fulfill traditional roles related to childcare and household responsibilities while participating in the labor force. Despite limited data, breastfeeding rates after returning to work average 25% and vary widely around the world [4]. While economic factors play a role, cultural influences strongly shape policies and workplace support.

In addition, cultural beliefs and societal stereotypes strongly influence breastfeeding decisions [5]. In some cultures, breastfeeding in public is still stigmatized, discouraging mothers from practicing it outside the home. In contrast, some traditions promote formula feeding as a sign of modernity or affluence, leading many mothers to choose formula feeding despite being aware of the benefits of breastfeeding. A narrative analysis study conducted in the United Kingdom [6] examined the factors influencing the breastfeeding experience of first-time mothers. The authors identified two distinct groups: those who stopped breastfeeding earlier than intended (referred to as disappointed mothers) and those who continued breastfeeding despite challenges. These findings highlight how breastfeeding is not just an individual choice but is shaped by external influences, including cultural expectations, healthcare systems, and workplace policies. From a feminist perspective, this underscores the need for policies that both support breastfeeding and respect a woman’s right to choose without guilt or social judgment [7].

Family dynamics and access to accurate health information also influence breastfeeding decisions [8,9]. Studies suggest that women who receive strong family support, particularly from their partners and close relatives, are significantly more likely to breastfeed. In contrast, misinformation from family members or a lack of appropriate guidance from health professionals can discourage mothers from initiating breastfeeding or cause them to discontinue breastfeeding prematurely [10,11,12]. In some cases, the promotion of skin-to-skin contact and the immediate initiation of breastfeeding contribute to higher breastfeeding rates [13].

Finally, but no less importantly, in the global promotion of a “breastfeeding culture”, research has largely overlooked fathers’/partners’ perspectives on their role in breastfeeding. Existing studies focus on their support for mothers and their attitudes toward breastfeeding but rarely explore their own experiences [14]. Breastfeeding offers significant health benefits for both infants and mothers. Despite global health recommendations advocating for exclusive breastfeeding during the first six months of life, many regions continue to experience suboptimal breastfeeding rates. Various factors contribute to this issue, including personal, sociocultural, economic, and healthcare-related barriers. While some research has explored programs involving fathers and partners, these studies primarily focus on enhancing breastfeeding promotion rather than deeply understanding the specific roles these individuals play in breastfeeding practices. This systematic review aims to examine the main barriers to breastfeeding by synthesizing evidence from studies published between 1995 and 2025, providing a comprehensive understanding of the factors that influence breastfeeding behaviors. By identifying and addressing these barriers, policymakers and healthcare providers can develop targeted interventions to improve breastfeeding success rates worldwide.

## 2. Materials and Methods

### 2.1. Research Question

This study employs a systematic review in accordance with the PRISMA (Preferred Reporting Items for Systematic Reviews and Meta-Analyses) [15] guidelines to systematically identify, appraise, and synthesize peer-reviewed literature that investigates the neural mechanisms that influence the perception of antagonistic characters. The PECO (population, exposure, comparator, outcome) framework was used to structure the research question and guide the study selection process. This systematic approach ensured a clear and objective assessment of the evidence related to barriers influencing breastfeeding attitudes and their impact on breastfeeding prevalence and success.

- Population (P): New mothers, breastfeeding.
- Exposure (E): Barriers influencing attitudes toward breastfeeding, including sociocultural norms, workplace policies, healthcare support, misinformation, and stigma.
- Comparator (C): This was considered optional. The comparator included situations where these barriers were absent or had a reduced impact, allowing for a comparison of breastfeeding outcomes in different environments.
- Outcome (O): The primary outcomes analyzed were breastfeeding prevalence, exclusive breastfeeding rates, breastfeeding success, initiation, continuation, and overall outcomes.

Based on the study design and the PECO framework, the research question could be formulated as “What are the key barriers influencing attitudes toward breastfeeding among new mothers, and how do these barriers impact breastfeeding prevalence, success, and continuation?” The review is registered in PROSPERO (2024) under the reference number CRD42024538998.

### 2.2. Search Strategy

To identify relevant studies, a literature search was conducted in EBSCOhost, including all its available databases, ensuring a broad and interdisciplinary approach to neuroscientific and narrative-related research. The search was performed using the following Boolean search syntax:

(“infant nutrition” OR “breastfeeding” OR “new mothers” OR “lactating women”) AND (“barriers” OR “challenges” OR “obstacles” OR “attitudes” OR “difficulties” OR “stigma” OR “social influences” OR “workplace” OR “cultural” OR “health support”) AND (“breastfeeding prevalence” OR “exclusive breastfeeding rates” OR “breastfeeding success” OR “breastfeeding initiation” OR “breastfeeding continuation” OR “breastfeeding outcomes”).

The following EBSCOhost databases were included in the search: (i) PsycINFO; (ii) MEDLINE; (iii) Academic Search Complete; (iv) Communication and Mass Media Complete; (v) ERIC; (vi) SocINDEX; and (vii) Cinahl.

### 2.3. Inclusion and Exclusion Criteria

This systematic review includes studies focusing on new mothers or lactating women and the barriers, challenges, or influencing factors affecting breastfeeding covering studies published between 2003 and 2025. Eligible studies must report on breastfeeding prevalence, exclusive breastfeeding rates, breastfeeding success, initiation, continuation, or related outcomes. Peer-reviewed quantitative, qualitative, and mixed-method studies, as well as systematic reviews and meta-analyses, are considered. To ensure relevance, only studies published within the last 30 years are included. The review considers research conducted in diverse cultural and socioeconomic settings and published in English or other specified languages.

Studies are excluded if they focus on pregnant women, non-lactating individuals, or unrelated populations such as fathers or healthcare providers. Research that does not specifically examine breastfeeding barriers or lacks relevant outcomes is also excluded. Additionally, opinion pieces, editorials, conference abstracts, and low-quality studies are not considered. Articles published more than 30 years ago are excluded unless they provide essential background knowledge.

### 2.4. Data Extraction and Analysis

Two independent reviewers screened the results. In cases where there was disagreement regarding the inclusion of a study (four conflicting cases), a third independent reviewer was consulted to make the final decision. To facilitate the screening process and enhance the rigor and efficiency of the systematic review, Rayyan software (https://new.rayyan.ai/ accessed on 22 March 2025) was employed. In this way, and after conducting the database search, all retrieved articles were screened for relevance based on their title and abstract, with only those meeting the inclusion criteria advancing to a full-text review. During this process, key data were systematically extracted and analyzed, including study details (authors, year, and journal), sample size and demographics (age, gender, and participant characteristics), and breastfeeding-related factors (barriers, cultural influences, workplace policies, and healthcare support).

### 2.5. Risk of Bias and Quality Assessment

Risk of bias and methodological quality were assessed using a structured adaptation of the Newcastle–Ottawa Scale (NOS), specifically tailored for the appraisal of observational studies in breastfeeding research. This modified tool evaluated selection bias, methodological rigor, and statistical appropriateness and has been previously used in similar systematic reviews. Studies were categorized as having low, moderate, or high risk of bias based on consensus by two independent reviewers

## 3. Results

**Figure 1:**
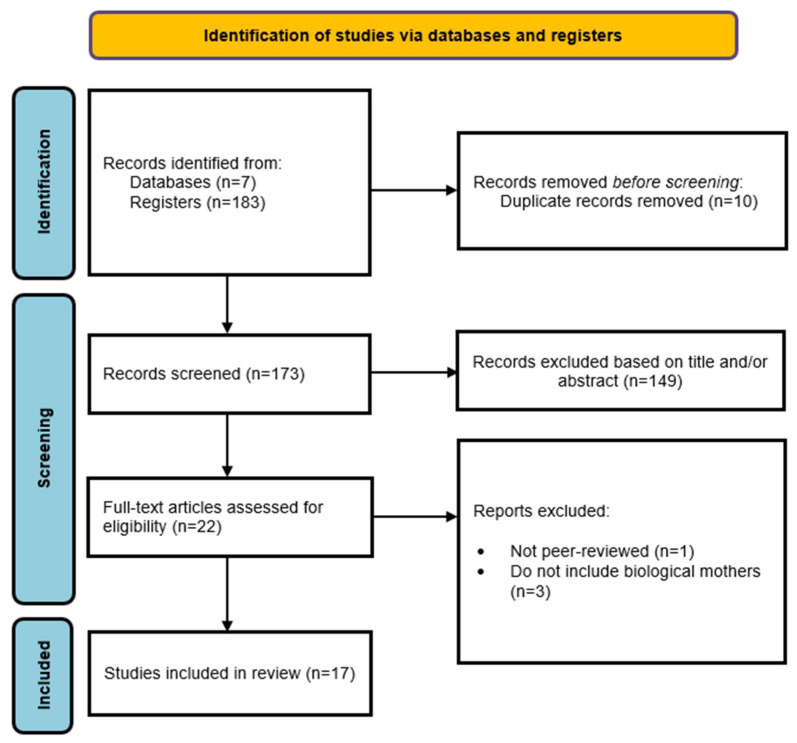
PRISMA flow diagram for systematic review: identification, screening, eligibility, and inclusion of studies in the current study.

Figure 1 presents the PRISMA (Preferred Reporting Items for Systematic Reviews and Meta-Analyses) flow diagram, which illustrates the step-by-step screening process that led to the inclusion of 17 studies in the review. The diagram provides a comprehensive overview of the selection process, starting with the total number of records initially identified through systematic searches across multiple electronic databases. This initial pool reflects the breadth of sources consulted to ensure a wide coverage of relevant literature. Following this, the diagram indicates the number of duplicate records that were identified and subsequently removed to avoid redundancy in the analysis.

The next stage presented in the diagram is the screening of titles and abstracts, where records were assessed against the predefined eligibility criteria. At this point, studies that did not meet the inclusion criteria were excluded, narrowing down the pool to the most relevant publications. The diagram then moves on to the full-text review stage, showing how many studies were retrieved and evaluated in depth for eligibility. It also specifies the number of studies excluded at this stage, accompanied by explicit reasons for their exclusion (e.g., methodological limitations, irrelevant outcomes, or insufficient data). Finally, the flow diagram highlights the number of studies that successfully met all inclusion criteria and were incorporated into the systematic review. This visual representation not only clarifies the rigorous selection process but also enhances transparency, allowing readers to understand how the final sample of studies was determined and ensuring the replicability of the review process.

Table 1 presents an overview of the 17 studies included in this systematic review, summarizing key details such as the main goals, methodologies, and outcomes of each study. This table allows for a quick comparison of the research objectives and approaches as well as the primary findings related to breastfeeding barriers and outcomes.

More precisely, multifactorial barriers to breastfeeding across diverse contexts and populations were identified. Key challenges identified include clinical and physiological barriers, such as biomechanical sucking difficulties in infants [16] and delayed lactogenesis among mothers of preterm infants [19]. Structural and workplace-related obstacles are also prominent, exemplified by the difficulties faced by mothers in healthcare settings, where inadequate lactation spaces, a lack of protected time, and unsupportive institutional cultures hinder breastfeeding continuation [27,30]. In addition, sociocultural barriers emerge as significant factors, including the absence of partner or family support, negative attitudes toward public breastfeeding, and the stigma associated with certain practices, such as exclusive formula feeding among HIV-positive women [25,32]. Collectively, these findings highlight the complex, interconnected nature of breastfeeding barriers, underscoring the need for comprehensive interventions at clinical, social, and structural levels to promote and sustain breastfeeding in varied settings.

**Figure 2:**
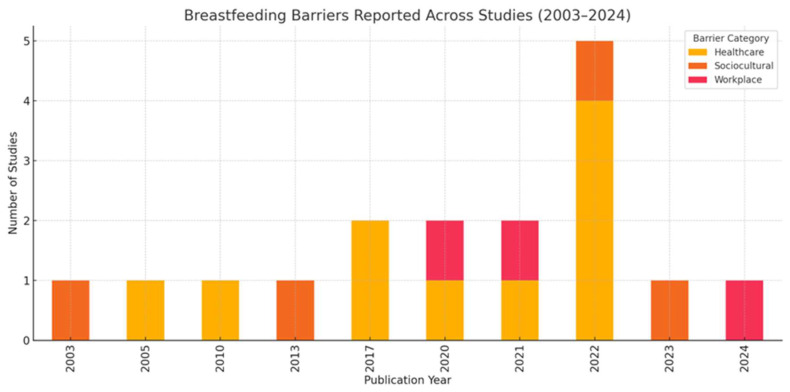
Manuscripts included in the review on a 10-year scale classified by category (healthcare, sociocultural, and workplace).

Figure 2 depicts the barriers to breastfeeding across a 10-year scale, categorized by barrier type (healthcare, sociocultural, workplace), based on the studies reviewed.

Table 2, on the other hand, focuses on assessing the risk of bias in the studies included in the review. This table evaluates the methodological quality of each study, considering factors such as sample size, study design, and potential sources of bias.

## 4. Discussion

Breastfeeding is widely recognized for its health benefits to both infants and mothers. However, despite global recommendations, breastfeeding rates remain suboptimal in many regions. This systematic review explores the barriers to breastfeeding, categorizing them into personal, sociocultural, economic, and healthcare-related factors. By analyzing peer-reviewed literature, this review identifies common challenges that hinder breastfeeding initiation and continuation.

A common theme across the studies is the influence of maternal health, infant conditions, and external factors like healthcare practices and social support on breastfeeding success. For example, the study by Herzhaft-Le Roy et al. [16] found significant improvements in sucking ability with osteopathic treatment, indicating the potential for non-pharmacological interventions in managing breastfeeding issues. However, other studies [18,26] revealed that while interventions may help to a degree, issues like poor breastfeeding technique or delayed lactogenesis remain persistent challenges for many mothers.

Specific risks highlighted include a lack of counseling and support from healthcare professionals, which can lead to difficulties in initiating and maintaining breastfeeding. For instance, inadequate lactation counseling has been associated with the early cessation of breastfeeding due to unresolved latching issues and maternal concerns about milk supply. Additionally, workplace-related challenges, such as insufficient maternity leave, a lack of private spaces for breastfeeding, and inflexible work schedules, have been identified as significant barriers that contribute to the early discontinuation of breastfeeding among employed mothers.

The studies also shed light on the sociocultural and psychological barriers to breastfeeding, with significant findings in research by Toro, Obando, and Alarcón [32] and Garti et al. [20] highlighting the critical role of social support networks in maintaining breastfeeding. In contrast, the challenges of maternal guilt and work-life balance, as seen in Moulton et al. [26] and Peters et al. [29], suggest that societal expectations and workplace policies significantly affect breastfeeding practices. Similarly, the increasing use of virtual lactation support, explored by different authors [8,24], reveals a shift in how breastfeeding guidance is delivered, particularly in the context of COVID-19, yet the effectiveness of such services requires further evaluation due to their limitations in providing hands-on assistance.

Results were also organized by barrier category (healthcare-related, sociocultural, workplace-related) and publication year. Healthcare-related barriers were consistently reported across the entire time frame, reflecting their persistent influence on breastfeeding outcomes. Sociocultural barriers appeared intermittently, with an increase in recent years, suggesting growing recognition of the role of social support, cultural norms, and stigma. Workplace-related barriers were predominantly reported in studies published after 2010, aligning with increasing research attention to maternal employment and lactation challenges in professional settings. This temporal visualization highlights both the enduring and emerging challenges mothers face in breastfeeding, underscoring the need for multi-level interventions that address healthcare, workplace, and sociocultural dimensions simultaneously.

The studies under review also highlight the importance of addressing lactation problems in specific populations, such as mothers using Assisted Reproductive Technology or those with preterm infants, where breastfeeding challenges were reported to be more severe. For instance, Dong et al. [19] identified that delayed lactogenesis II was prevalent among mothers of preterm infants, which underscores the need for targeted interventions for these high-risk groups.

In terms of study quality, most of the included studies were subject to a moderate risk of bias, particularly due to sample size and methodological limitations. Some studies, like [21,24], had less bias and stronger data, making their conclusions more trustworthy. This variability in study quality emphasizes the need for more rigorous research with larger, more diverse samples to ensure the generalizability of findings. Nevertheless, one should bear in mind that the quality assessment used a modified version of the Newcastle–Ottawa Scale, adapted for breastfeeding-related observational studies. While this tool has not undergone formal validation, it allowed for a consistent appraisal of selection and performance bias across heterogeneous study designs.

It should be noted that little attention is given in the literature to the role of partners, particularly in how their support or lack thereof can influence breastfeeding success [14]. The absence of a broader focus on partners’ support and cultural influences leaves a gap in understanding the full spectrum of factors that affect breastfeeding practices.

While this review provides valuable insights into the various challenges and interventions related to breastfeeding, it is not without limitations. Many of the studies included in the review had small, homogenous sample sizes, which limits the generalizability of their findings. The variability in research methodologies, from randomized controlled trials to qualitative interviews further complicates comparisons across studies. Additionally, several studies lacked long-term follow-up, which makes it difficult to assess the sustained impact of interventions. Future research should focus on larger, more diverse populations and employ longitudinal designs to evaluate the long-term effectiveness of breastfeeding interventions. Future research should explore not only interventions but also the mechanisms by which cultural, workplace, and social dynamics shape breastfeeding practices. Hypotheses for future studies might include how integrated workplace policies and paternal involvement affect breastfeeding duration or how combined in-person and virtual support can improve breastfeeding outcomes.

## 5. Conclusions

This systematic review aimed to identify the key barriers influencing breastfeeding attitudes, initiation, and continuation. The findings demonstrate that breastfeeding practices are shaped by multifactorial barriers across personal, clinical, sociocultural, and workplace domains. Healthcare-related challenges, including insufficient counseling and inconsistent lactation support, were among the most pervasive. Sociocultural influences—such as stigma, misinformation, and a lack of partner or family support—also played a significant role. Workplace constraints further compounded these difficulties, particularly among employed mothers.

To improve breastfeeding outcomes, interventions must adopt a multidimensional approach that integrates healthcare reform, workplace accommodations, and culturally sensitive education. Future research should investigate the longitudinal effects of combined in-person and virtual lactation support and examine the impact of partner involvement and workplace policies on breastfeeding duration.

## Figures and Tables

**Table 1:** Manuscripts included in the review.

Study	Study Objective	Participants	Methodology	Results
[16]	To evaluate the efficacy of an osteopathic treatment combined with lactation consultations for infants with biomechanical sucking difficulties	97 mother–infant dyads with biomechanical sucking difficulties, younger than 6 weeks	Randomized controlled trial, blinded for participants, assessing sucking ability using the LATCH tool and maternal pain through a visual analog scale	The group treated with osteopathy showed significant improvements in sucking ability compared to the control group, but there were no differences in nipple pain.
[17]	To assess the challenges of lactation and breastfeeding among mothers who conceived through ART (Assisted Reproductive Technology)	196 mothers who gave birth after an ART pregnancy at SIMS Hospital, Chennai, India	Questionnaire-based survey administered to mothers at 3 months postpartum, analyzed using multivariate logistic regression	Many mothers experienced breastfeeding difficulties, such as failure to lactate or latching issues. Depression and multiple births were associated with greater difficulties.
[18]	To assess the impact of an intervention on breastfeeding technique on the rate of exclusive breastfeeding and related problems	211 mothers and babies in a maternity hospital in Brazil	Randomized clinical trial with a single intervention in the maternity ward, breastfeeding evaluation at birth, 7 days, and 30 days	No significant improvement in breastfeeding technique quality, although there was a slight tendency for better latch in the intervention group.
[19]	Investigate lactation status and breastfeeding challenges in mothers of preterm infants	124 mothers of preterm infants (26 May–31 October 2018, China); 70 analyzed	Questionnaires at four time points: day 7 postpartum, infant discharge, 2 weeks post-discharge, and 3 months corrected age. Logistic regression and ROC analysis were used.	51.4% had delayed lactogenesis II, with older age and first live birth as predictors. Challenges included insufficient milk, feeding complications, and poor technique.
[20]	To evaluate infant feeding practices and their association with malnutrition in young children in Ghana	403 mothers/caregivers with children aged 6 to 23 months attending child welfare clinics in Techiman, Ghana	Analytical cross-sectional design, simple random sampling from 8 health centers, data collection through 24 h dietary recall and anthropometric measurements	Delayed breastfeeding initiation and bottle feeding were associated with acute malnutrition, while feeding challenges were linked to chronic malnutrition.
[21]	To examine the relationship between lactation problems and benign/malignant breast disease	308 patients referred to two breast clinics in Tehran (2008–2011)	Standard questionnaire on breastfeeding issues, classified into 3 groups: breastfeeding without problems, unwillingness to breastfeed, and insufficient milk.	Unwillingness to breastfeed and breast problems like mastitis were significantly linked to both benign and malignant breast diseases (*p* < 0.01), while insufficient milk showed no association.
[22]	Explore the feasibility, benefits, challenges, and patient satisfaction with virtual lactation services in Ontario	177 survey responses	Online survey assessing patient satisfaction with virtual lactation and pediatrician consultations	86.44% were satisfied with the virtual lactation services. Satisfaction was higher among first-time mothers aged 26–35, with high school or undergraduate education, and those living in the Greater Toronto Area (GTA).
[23]	Explore lactation support providers’ perspectives on in-person and telehealth consultations and the impact of COVID-19	14 lactation support providers in Massachusetts, ages 36–68	Qualitative descriptive study with online surveys and virtual interviews	Main themes: common client questions, consultation topics, telehealth vs. in-person facilitators and barriers, best practices, and COVID-19 adaptations. COVID-19 led to a shift to telehealth, which had advantages like scheduling flexibility but lacked hands-on assistance. In-person consultations allowed for physical assessments, but unsupportive family members were a barrier.
[24]	Examine the success of Brazil in supporting HIV-positive women to exclusively formula feed (EFF) their infants	30 HIV-positive women in Salvador, Brazil	In-depth interviews about attitudes, practices, and challenges related to EFF	Most women adhered to EFF, motivated by postpartum counseling about HIV transmission risks. Challenges included reconciling breastfeeding as a maternal duty, stigma, and unexpected financial burdens. EFF has contributed to declining vertical HIV transmission, but further support services are needed.
[25]	Determine incidence and risk factors for early lactation problems among mother–infant pairs in Lima, Peru	171 primiparous mothers with healthy, single, term infants	Data collected on day 0 (hospital) and day 3 (home visit); breastfeeding behavior assessed using the Infant Breastfeeding Assessment Tool	SIBB prevalence was 52% on day 0 and 21% on day 3; associated with male infant gender, <8 breastfeeds in the first 24 h, and <39 weeks gestational age. Delayed lactogenesis occurred in 17%, linked to Apgar score < 8. Excessive neonatal weight loss (10%) was associated with maternal overweight and C-section delivery. Early lactation issues were influenced by delivery mode and breastfeeding frequency.
[26]	Explore social and environmental conditions affecting workplace lactation in emergency departments	24 individuals in EDs with recent return-to-work experience after childbirth (21 faculty, 12 trainees, 3 nurses)	Constructivist grounded theory; 36 unique lactation experiences analyzed using thematic coding	Three themes emerged: (1) emergency medicine culture, (2) workplace lactation policies, and (3) support for workplace lactation. Cultural barriers persist despite policies and support, indicating a need for broader cultural change in EDs to fully support lactating individuals.
[27]	Explore stressors related to workplace lactation spaces for individuals in emergency medicine	40 medical students, residents, nurses, fellows, and faculty	Qualitative study with thematic analysis of interviews about post-pregnancy return-to-work experiences	Lactation spaces contribute to stress due to both tangible (e.g., access to computers) and intangible factors (e.g., privacy, time management). Participants expressed a desire to work while pumping to balance dual roles. Thoughtfully designed lactation spaces can empower lactating clinicians and improve workplace satisfaction.
[28]	To identify problems of lactation among postnatal mothers	30 postnatal mothers	Quantitative descriptive research through a questionnaire	Many mothers experience physical challenges. A significant number of mothers—around 100 in some studies—experience insufficient milk supply during breastfeeding.
[29]	To assess issues surrounding breastfeeding during graduate medical training for female residents in the US	312 female residents from US residency programs (respondents from 2017 onwards)	National cross-sectional survey using Qualtrics; summary statistics and free-text responses	21% had access to usable lactation rooms and 60% lacked a place to store breast milk; 73% reported that residency limited their lactation ability, and 37% stopped breastfeeding early; 40% felt guilty due to faculty or co-residents, and 56% experienced mental health impacts from breastfeeding difficulties.
[30]	To understand barriers and facilitators experienced by lactation professionals (LPSs) in Appalachia when providing services to families	89 LPSs (survey); 20 LPSs (interviews)	Mixed-methods explanatory sequential design: survey followed by semi-structured interviews	Barriers included challenges with other healthcare providers, hospital practices, and family support. Facilitators included social support from other LPSs, and social media/telehealth was both helpful and problematic. LPSs also identified the need for additional training in various areas, including substance use, mental health, and support.
[31]	To compare infant feeding attitudes of parents of breastfed versus formula-fed infants	108 couples (pregnant women and partners)	Survey using the Iowa Infant Feeding Attitude Scale	Parents of breastfed infants had more positive attitudes and greater knowledge about breastfeeding’s benefits. Fathers were more likely to disapprove of public breastfeeding. Mothers of formula-fed infants held misconceptions about breastfeeding, such as the effects of alcohol consumption.
[32]	To explore the social valuation of breastfeeding and difficulties influencing early weaning in Chile	35 breastfeeding mothers	Semi-structured interviews with qualitative phenomenological approach, grounded theory analysis	Social support networks, particularly from partners and family, are crucial for maintaining exclusive breastfeeding (EBF). Weaning occurred when these networks were unavailable. The breastfeeding process is complex and influenced by social factors that can either support or hinder continued breastfeeding.

**Table 2:** Risk of bias.

Study	Selection Bias (Sample Size and Diversity)	Performance Bias (Methodological Rigor)	Overall Risk of Bias
[16]	Moderate (randomized controlled trial, but could be limited by sample size and characteristics of dyads)	Low (randomized controlled trial, blinded)	Low
[17]	Moderate (196 mothers, but limited to one hospital and ART pregnancies)	Moderate (questionnaire-based, though analyzed with multivariate logistic regression)	Moderate
[18]	Low (large sample size in a maternity hospital in Brazil)	Moderate (single intervention and limited to a specific hospital setting)	Moderate
[19]	Moderate (124 mothers, but preterm infants are a specific group)	Moderate (questionnaires and logistic regression, may have sampling biases)	Moderate
[20]	Low (403 mothers/caregivers, random sampling from health centers)	Moderate (24 h recall and anthropometric measurements, but cross-sectional design)	Moderate
[21]	Low (308 patients from two clinics)	Low (standard questionnaire, large sample)	Low
[22]	Low (177 responses from diverse mothers in Ontario)	Low (online survey, well-structured)	Low
[23]	Moderate (small sample size of 14 lactation support providers)	Low (qualitative study with interviews, rigorous analysis)	Moderate
[24]	Low (30 HIV-positive women, though limited to Brazil)	Low (in-depth interviews, qualitative approach)	Low
[25]	Low (171 primiparous mothers, single maternity hospital)	Low (clinical data, breastfeeding assessment)	Low
[26]	Low (24 participants from emergency departments)	Moderate (thematic coding, qualitative study)	Moderate
[27]	Low (40 participants from emergency medicine)	Moderate (thematic analysis of interviews, qualitative)	Moderate
[28]	Low (30 postnatal mothers)	Moderate (descriptive quantitative research, questionnaire)	Moderate
[29]	Moderate (312 female residents, diverse training programs but no further details)	Moderate (survey-based, potential non-response bias)	Moderate
[30]	Moderate (89 surveys, 20 interviews, mixed methods)	Moderate (survey and interview methods, may lack representativeness)	Moderate
[31]	Low (108 couples, adequate sample size)	Low (survey, consistent method used)	Low
[32]	Moderate (35 breastfeeding mothers, small sample size)	Low (semi-structured interviews, rigorous analysis)	Moderate

## Data Availability

No new data were created or analyzed in this study.

## Abbreviations

The following abbreviations are used in this manuscript:

PRISMA	Preferred Reporting Items for Systematic Reviews and Meta-Analyses
PECO	Population, Exposure, Comparator, Outcome
NOS	Newcastle–Ottawa Scale
ART	Assisted Reproductive Technology
EFF	Exclusively formula feed
